# Interruptive Versus Noninterruptive Clinical Decision Support: Usability Study

**DOI:** 10.2196/12469

**Published:** 2019-04-17

**Authors:** Saul Blecker, Rishi Pandya, Susan Stork, Devin Mann, Gilad Kuperman, Donna Shelley, Jonathan S Austrian

**Affiliations:** 1 Department of Population Health New York University School of Medicine New York, NY United States; 2 Department of Medicine New York University School of Medicine New York, NY United States; 3 Memorial Sloane Kettering Cancer Center New York, NY United States

**Keywords:** clinical decision support, hospital, electronic health records

## Abstract

**Background:**

Clinical decision support (CDS) has been shown to improve compliance with evidence-based care, but its impact is often diminished because of issues such as poor usability, insufficient integration into workflow, and alert fatigue. Noninterruptive CDS may be less subject to alert fatigue, but there has been little assessment of its usability.

**Objective:**

This study aimed to study the usability of interruptive and noninterruptive versions of a CDS.

**Methods:**

We conducted a usability study of a CDS tool that recommended prescribing an angiotensin-converting enzyme inhibitor for inpatients with heart failure. We developed 2 versions of the CDS: an interruptive alert triggered at order entry and a noninterruptive alert listed in the sidebar of the electronic health record screen. Inpatient providers were recruited and randomly assigned to use the interruptive alert followed by the noninterruptive alert or vice versa in a laboratory setting. We asked providers to “think aloud” while using the CDS and then conducted a brief semistructured interview about usability. We used a constant comparative analysis informed by the CDS Five Rights framework to analyze usability testing.

**Results:**

A total of 12 providers participated in usability testing. Providers noted that the interruptive alert was readily noticed but generally impeded workflow. The noninterruptive alert was felt to be less annoying but had lower visibility, which might reduce engagement. Provider role seemed to influence preferences; for instance, some providers who had more global responsibility for patients seemed to prefer the noninterruptive alert, whereas more task-oriented providers generally preferred the interruptive alert.

**Conclusions:**

Providers expressed trade-offs between impeding workflow and improving visibility with interruptive and noninterruptive versions of a CDS. In addition, 2 potential approaches to effective CDS may include targeting alerts by provider role or supplementing a noninterruptive alert with an occasional, well-timed interruptive alert.

## Introduction

Clinical decision support (CDS) systems have been shown to improve provider compliance with evidence-based cardiovascular care in the inpatient hospital setting [1,2]. Nonetheless, the effectiveness of CDS interventions is frequently diminished because of issues such as poor usability, insufficient integration into provider workflow, and alert fatigue [3-6]. These limitations are particularly problematic in the inpatient setting, where providers are concurrently caring for numerous patients with urgent needs located in multiple locations.

CDS alert fatigue is frequently related to the fact that interruptive alerts force providers to notice or respond to the CDS in the middle of their other tasks. Noninterruptive CDS tools, which do not require stoppage of other electronic health record (EHR) activity, may be less subject to alert fatigue [6,7]. A number of studies have demonstrated that noninterruptive alerts can increase provider compliance with care measures such as venous thromboembolism prevention in the inpatient setting [8,9], yet this type of alert is generally perceived as less successful at changing provider behavior compared with interruptive alerts [6,7]. Nonetheless, the few studies that compare the relative uptake of interruptive and noninterruptive alerts have not consistently shown interruptive alerts to be superior [10,11]. Finally, despite assumptions that noninterruptive alerts have less effect on workflow [6,7], there has been little evaluation of the relative usability of interruptive and noninterruptive alerts. Although prior studies have evaluated the usability of either interruptive or noninterruptive alerts [9,12,13], we are unaware of studies that have compared the usability of these 2 implementation approaches. Information about their relative usability can help inform developers of CDS about the relative advantages of interruptive and noninterruptive alerts. In addition, evaluation of usability of these 2 CDS implementation approaches may be particularly useful in the inpatient setting, where providers frequently deal with competing demands and interruptions to workflow.

Usability relates to the extent a system will allow end users to complete a task in an effective, timely, and satisfactory way [14,15]. Usability testing draws on the principles of human-computer interaction to evaluate the usability of a system and is considered best practice in the development of EHRs and related systems in health care [14-17]. The purpose of this study was to pilot test the comparative usability of an interruptive version versus a noninterruptive version of an inpatient-focused CDS.

## Methods

### Study Design

We conducted a usability study of a CDS tool that recommended prescribing an angiotensin-converting enzyme (ACE) inhibitor for inpatients with heart failure. The setting was NYU Langone Health, an urban academic medical center with approximately 3000 hospitalizations with a diagnosis of heart failure annually [18]. We recruited individual health care providers to use the tool in a laboratory setting and provide feedback on usability. We created 2 versions of the CDS: one an interruptive alert and the other a noninterruptive alert. We then randomly assigned providers to use the interruptive alert followed by the noninterruptive alert or vice versa; we randomly assigned the order for presentation to minimize the effect that using one version of the alert may have on feedback on the second version of the alert. Order assignment was based on random number generation. Following usability assessment, we conducted a brief semistructured interview for additional feedback.

### Subjects and Recruitment

We included individual health care providers who care for and write medication orders for hospitalized adult patients. We excluded providers who do not write inpatient medication orders. We identified and recruited potential participants through sending emails to relevant department listservs, colleagues of study team members, and suggestions from prior interviewees. We used a purposive sampling framework: we invited participants to ensure a range of services, including medicine and surgery, and provider types, including attending physicians, resident physicians, nurse practitioners (NPs), and physician assistants. However, we stopped recruiting attending physicians after the first interview, in which the attending physician reported exclusively relying on residents, NPs, and physician assistants to write orders. Recruitment continued until a range of services and provider types was achieved and thematic saturation was reached. Participants received a US $25 gift card after completion of the interview.

### Clinical Decision Support Intervention Description

We developed 2 versions of the CDS intervention that had similar triggering actions but varied in their format for presentation. The CDS interventions were built within the sandbox testing environment of the EHR at NYU Langone Health, Epic (Epic Systems, Verona, Wisconsin). The initial development was led by the study team using input from clinical leadership and based on standard Epic templates. Development was informed by interviews with end-user providers [19]. Both versions of the CDS ultimately presented a dialogue box that informed providers that the patient had a reduced ejection fraction (EF), was not on an ACE inhibitor or angiotensin receptor blocker, and that these medications are potentially beneficial to patients with this condition [20,21]. Usual contraindications were explained, and recent values for blood pressure, estimated glomerular filtration rate (eGFR), and potassium were listed [20]. Providers were given the options to order an ACE inhibitor (lisinopril 5 mg daily), report a contraindication, or simply dismiss the CDS.

**Figure 1:**
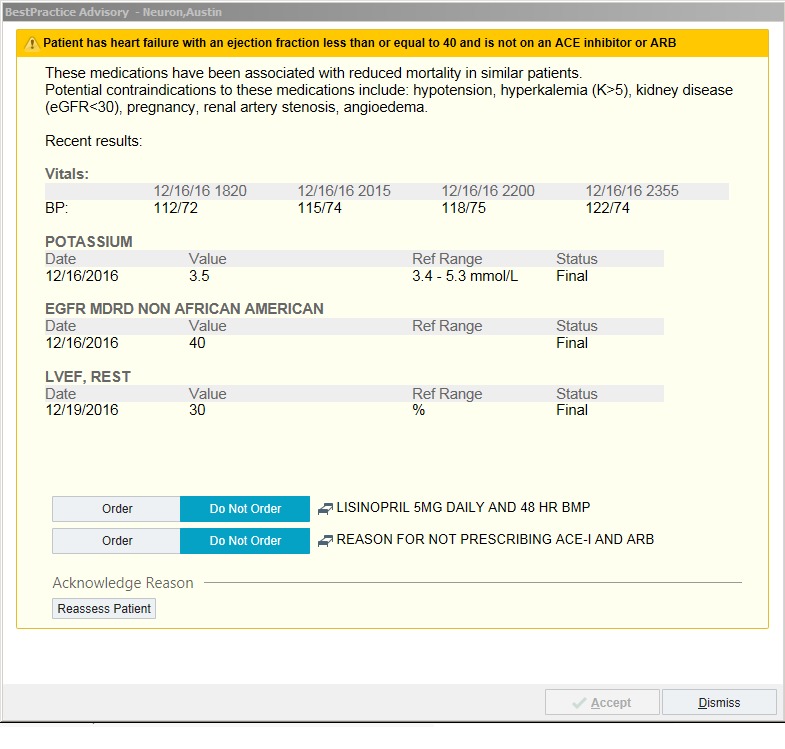
Screenshot of clinical decision support used in usability testing: interruptive version of clinical decision support. Source: Epic Systems Corporation; used with permission. ACE: angiotensin-converting enzyme; ARB: angiotensin receptor blocker; BP: blood pressure; eGFR: estimated glomerular filtration rate; LVEF: left ventricular ejection fraction.

**Figure 2:**
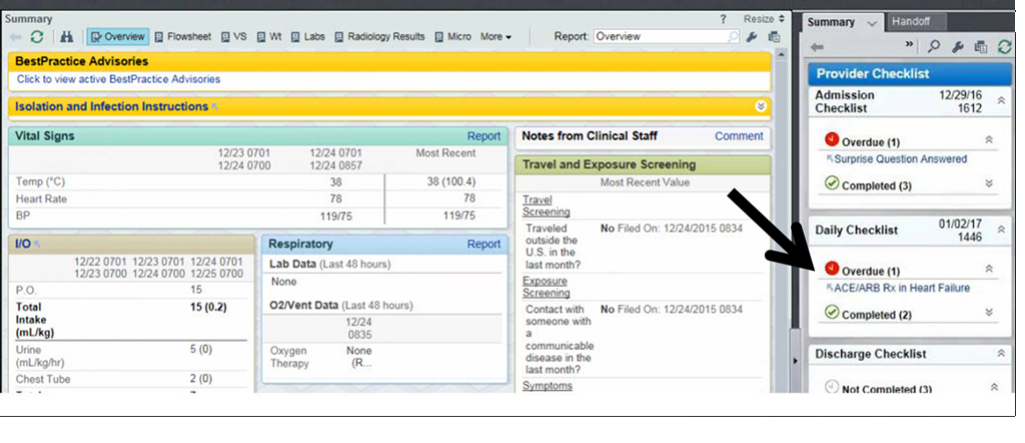
Screenshot of clinical decision support used in usability testing: location of noninterruptive version of clinical decision support, highlighted by the arrow. Clicking the link in the noninterruptive alert would take the user to a screen similar to the interruptive alert. Source: Epic Systems Corporation; used with permission. ACE: angiotensin-converting enzyme; ARB: angiotensin receptor blocker; BP: blood pressure.

The format of the first alert was interruptive, in which the CDS dialogue box popped up at the time of order entry (Figure 1). The second version was a noninterruptive link that was located in a sidebar *checklist report* (Figure 2). This sidebar was part of the usual EHR display, and the interruptive alert was present in the sidebar until the CDS criteria were satisfied. Selecting the hyperlink in the sidebar led to the presentation of the same CDS dialogue box as in the interruptive alert.

### Usability Testing

We first obtained verbal consent for participation and audio recording. We then provided participants with a clinical scenario in which they were caring for a patient who had heart failure with a reduced EF and who was principally hospitalized for another condition related to the provider’s specialty, such as pneumonia, stroke, or surgery. Providers were advised that they were to proceed with opening the patient’s chart and ordering morning laboratory tests. For providers assigned to the interruptive alert, the alert would trigger once they initiated the process to order labs. Some providers who were first assigned to the noninterruptive alerts would see and attempt to work with the CDS as well; for those who had not noticed the noninterruptive alert after a few minutes of charting, we also directed them to the CDS. While working with the CDS, providers were asked to *think aloud* [17,22]. In the think-aloud method, users verbalize their thoughts and offer feedback while interacting with the CDS to identify usability issues.

After working through the first alert, we asked providers about navigation, content, ease of use, fit into workflow, and suggestions. We then performed usability testing on the other version of the alert using the same procedure. Providers were then asked about usability of the second version of the alert and the comparative advantages of each version of the CDS tool. Finally, we asked providers to complete a brief demographic survey.

### Qualitative Analysis

Audio recordings from usability testing were transcribed by a professional transcription service. Transcriptions were reviewed against recordings, with any mistakes corrected.

We used the constant comparative method to analyze the usability testing, which included audio recordings from both the think-aloud protocol and the semistructured interview questions. In this qualitative analysis technique based on grounded theory [23,24], we began with open codes that were progressively grouped and refined into code categories. At least 2 of 3 coders (SB, RP, and SS) independently coded each of the transcripts and then met to review codes and reach consensus on any disagreements. Throughout the coding process, coders also met regularly with the larger investigative team to review and refine the code list. We categorized codes as being related to the general CDS, the interruptive alert, and the noninterruptive alert. Emergent themes were informed by the CDS Five Rights CDS framework as well as by prior work in CDS usability testing [13,15,25]; the Five Rights framework postulates that CDS is most effective when the right information is delivered to the right person, through the right intervention format and the right channel, and at the right time in workflow. We grouped all codes into 1 of the 4 rights in the framework; no codes were related to the theme of right channel as the CDS was delivered exclusively through the EHR.

## Results

We conducted usability testing with 12 providers. Overall, 9 of these providers (75%) were on the medicine service; the remaining providers were in surgery or neurology (Table 1). Half of the providers self-identified as Asian. Furthermore, 7 providers were randomly assigned to test the usability of the interruptive alert followed by the noninterruptive alert; the remaining 5 providers started with the noninterruptive alert. Interview lengths ranged from 11 to 29 min.

We categorized codes from the usability testing, which combined the think-aloud interviews and the responses to semistructured questions, into 4 themes related to the CDS Five Rights. We defined some codes as related to the CDS in general and others as related to the interruptive or noninterruptive version of the CDS (Textbox 1 and Table 2).

### Right Information

Nearly all codes that related to the general CDS fit within the theme of right information and could generally be categorized as positives, negatives, or suggestions for the CDS. Positives about the CDS included that providers thought that the CDS would be helpful to alert them that the patient had heart failure and may not be on evidence-based therapy. They expressed that the reported contraindications in the text and reporting of the relevant vital signs and laboratory results were useful. Nonetheless, providers suggested adding the following elements: trends for laboratory results, creatinine results, summary of past ACE inhibitor use, and contraindications to ACE inhibitors. Concurrently, some providers gave negative feedback about too much information, which could impede workflow, as suggested by 1 first-year resident:

I don’t know if there’s a way to make it even more brief...there’s too much text...it was slowing me down.

The primary negative feedback related to usability stemmed from a lack of clarity on the slide button that allowed for options to *order* or *do not order* each of the ACE inhibitor or reason for not prescribing. A second-year resident made this suggestion:

It was a little confusing...I don’t know if there’s a way to make it to that so it’s order or not order the ACE, and then the second one [for] if you didn’t order the ACE.

**Table 1:** Characteristics of 12 providers participating in usability testing.

Characteristic		Statistics, n (%)
**Clinical service**		
	Medicine	9 (75)
	Neurology	1 (8)
	Surgery	2 (17)
**Clinical role**		
	Attending	1 (8)
	Resident	—^a^
	First-year resident	2 (17)
	Second-year resident	4 (33)
	Nurse practitioner	4 (33)
	Physician assistant	1 (8)
**Years in current role**		
	1-3	6 (50)
	4-10	4 (33)
	>10	2 (17)
Female		4 (33)
**Ethnicity**		
	Not Hispanic or Latino	11 (92)
	Missing	1 (8)
**Race**		
	White	3 (25)
	Black	1 (8)
	Asian	6 (50)
	Multiracial	2 (17)

^a^Not applicable.

### Right Person

Perceived role, a contributor to the theme of right person, also influenced whether providers found the general CDS tool to be useful. In particular, a number of providers felt that it was their responsibility to deliver evidence-based care, including for an ACE inhibitor in heart failure. However, some providers, including those on surgical services and those who perform cross-coverage duties, found the CDS to be outside of their scope of practice. These providers wished for the option to dismiss the CDS for themselves but not for other providers. In this approach, the CDS would only continue triggering providers whose perceived role was appropriate for the CDS recommendations.

The theme of right person also applied to each version of the alert. A second-year medicine resident preferred the noninterruptive alert because of their perceived role to conduct global reviews of their patients:

At the end of the day I look through every [patient’s checklist] as a [senior] resident. As [a first year resident] maybe not because I’m the one putting in all the orders.

A first-year resident felt the interruptive alert would be useful because:

In the acute setting, especially, Lisinopril might get missed until we discuss it during rounds, but then if you put that there as an alert for us to see. [If I am too busy to] order at that time I feel like I would write it down somewhere to keep myself...I keep a sheet with all the patients and to-dos for every patient...and I definitely won’t forget that because I know by the end of the day I want to check off all the boxes.

**Codes from usability testing categorized into themes based on the Clinical Decision Support Five Rights. Groups further based on the interruptive or noninterruptive version of alert or usability groups.**

*Right information*

- Content/usefulness
  - Alerts to best practice
  - Alerts to presence of heart failure
  - Labs and vital signs relevant
  - Contraindications to therapy useful
  - Wants a summary of current and prior medications
  - Wants lab or vital sign trends
  - No creatinine listed
  - Less information would be helpful
- Usability
  - Easy to locate relevant information
  - Difficulty or confusion with “order” versus “do not order” button
  - Does not notice the reason for not prescribing
  - Difficulty with ordering basic labs within clinical decision support

*Right person*

- General alert
  - Recommendation not within the perceived scope of practice
  - Responsibility to deliver evidence-based therapy
- Noninterruptive version of alert
  - Responsibility as a resident to address noninterruptive alerts

*Right time in workflow*

- Noninterruptive version of alert
  - Likes ability to address at a later time
  - Reviewing noninterruptive alerts part of workflow
- Interruptive version of alert
  - Prefers if delivered at right time in workflow
  - Impedes workflow
  - Wants option to address at a later time

*Right intervention format*

- Noninterruptive version of alert
  - Not always noticed
  - Flagged alerts increase visibility
  - Likes that can defer task to another provider
  - May defer and then forget about alert
  - Would notice and address noninterruptive alert
  - Would prefer alerts in a more visible location
- Interruptive version of alert
  - More noticeable
  - Pays less attention to content of interruptive alert
- Either version
  - Prefers combination of interruptive and noninterruptive alert

**Table 2:** Example quotations from usability testing by an interruptive or noninterruptive version of the clinical decision support.

Theme and clinical decision support version		Example code	Example quotation
**Right information**			
	General (content)	Wants lab or vital sign trend	“It’s better to have a trend...I’m more comfortable ordering this because I see those three times patient is very stable”
	General (usability)	Difficulty with “order” versus “do not order” button	“To me that’s a little counter-intuitive, but it could be that there’s other sections of [the EHR] where that’s how you document not doing something.”
**Right person**			
	General	Not within the perceived scope of practice	“I wouldn’t necessarily start a patient on a medication just because of my specialty.”
	Noninterruptive	Responsibility as a resident to address noninterruptive alerts	“I started using the provider checklist a little bit more especially as a resident when you’re reviewing things.”
**Right time in workflow**			
	Noninterruptive	Reviewing alert part of workflow	“At the end of the day I look through everyone, make sure...things are checked. Then I would notice things that are here.”
	Interruptive	Impedes workflow	“This one is a little bit more annoying because it will prevent me from doing what I wanna do.”
**Right intervention format**			
	Noninterruptive	Not always noticed	“If you hadn’t have told me that this was on the right-hand side, I never would have noticed it in the first place. Now that I see it here it's actually nice.”
	Interruptive	Pays less attention to content of interruptive	“When we get a lot of them we tend to just turn off—when I see it I just barely breeze right through it and just hit dismiss.”
	Either version	Combine interruptive and noninterruptive	“I don’t know if there’s any way to make it pop up if you haven’t reviewed the provider checklist by the end of the day.”

### Right Time in Workflow

A number of providers expressed that they preferred the noninterruptive version as it fit better within their workflow by not impeding current tasks. They expressed appreciation that they could address a noninterruptive alert at a later point, according to their own workflow. Some providers expressed that reviewing noninterruptive CDS tools was part of their current daily routine. Conversely, many providers agreed with an NP who described the interruptive CDS as *annoying* and impeded workflow. They requested the capability to defer the alert until a later time but did note that the interruptive version would be preferred if activated at the right time in their workflow.

### Right Intervention Format

Many providers said that they do not always notice noninterruptive alerts or that they defer these alerts and forget to return to them at a later time. Others found the location on the screen to have inadequate visibility or believed that there were too many flagged alerts on the screen, making the alert less noticeable. Indeed, over half of the providers (7/12) did not quickly notice the noninterruptive alert and were directed to its location on the screen; of these providers, 3 were initially assigned to the noninterruptive alert and 4 were initially assigned to the interruptive alert. Conversely, some providers found the noninterruptive alert flags to be readily visible and appreciated the ability to defer these alerts to another time and, if appropriate, to another provider. Providers found the interruptive alert to be more noticeable, which is why some preferred this format; concurrently, others said they would not pay attention to the interruptive alert, including 1 second-year resident:

I feel making it mandatory makes it like I'd pay attention to it less.

Given the noted trade-offs between the 2 versions of these alerts, some providers thought a combined version would be most useful. For instance, the noninterruptive alert could be available continuously but, if not utilized within a certain timeframe, would be enhanced with triggering of the interruptive alert, as described by 1 second-year resident:

You have to electively review the [noninterruptive alert], which everyone may or may not do...I don’t know if there’s any way to make it pop up if you haven’t reviewed by the end of the day or [another] time frame.

## Discussion

### Principal Findings

We found that many providers expressed annoyance in working with an interruptive CDS, primarily because it would interrupt workflow. A noninterruptive version of the CDS was appealing to providers, given that it could be accessed at any time in the workflow or seamlessly deferred to other providers. However, providers acknowledged that a noninterruptive alert was frequently not noticed and may not support clinical decision making unless integrated into routine workflow. One suggestion was to balance the 2 approaches by combining formats: supplementing a noninterruptive alert with an occasional, well-timed interruptive alert if uptake was insufficient. Given the reported trade-off of distraction and visibility between the interruptive and noninterruptive alerts, we intend on implementing both versions of the CDS in our hospital system to determine relative use and usability in clinical practice.

Although individual providers differed on their description of how each version of the alert would fit into their workflow, one of our key findings was that provider role seemed to be associated with the acceptability of the CDS format. In particular, some providers expressed that their role in residency training affected their preference for how the CDS was delivered. With the caveat that this small qualitative study was not powered to represent subgroups, we found that 1 first-year resident, whose role is primarily related to implementation of the care that is delivered in the hospital, tended to favor the interruptive CDS as it alerted this provider to another task to accomplish for the day. Conversely, more senior residents, whose role is defined by overseeing the delivery of care for patients, tended to favor the noninterruptive CDS. These residents felt that such a CDS could aid in their broad assessment of an individual patient’s care at the opportune time when performing such a review.

Our finding of a potential interaction between provider role and fit of CDS into workflow builds off prior studies examining provider characteristics and potential for uptake of CDS [26-28]. For instance, surveyed providers were more likely to report acceptance of a CDS if not behind in their work [28], and in secondary analysis, a CDS tool for respiratory symptoms was more likely to be used by resident providers as compared with attending providers [27]. The CDS Five Rights framework specifies the importance of both provider role and intervention format [25]. This framework has led to CDS systems designed to deliver different information for clinicians in different roles; for example, 1 CDS system included an alert to nurses if a patient had signs of early sepsis while concurrently offering a separate sepsis order set to providers [29]. Nonetheless, we are unaware of a CDS that was developed to specifically address the potential interaction between role and intervention format. Our data suggest an opportunity to increase CDS usability—ultimately with the goal of increasing uptake—by targeting providers who may find that a given format fits best within their clinical role. An example of this based on our preliminary findings could be that the interruptive alert targets first-year residents, whereas the noninterruptive alert targets senior residents; however, we would need to better survey residents before such an implementation.

One of the primary purposes of usability testing of a CDS tool is to adjust the tool based on end-user feedback [15]. We made some changes to our CDS during usability testing based on initial feedback, including incorporating additional trends in blood pressure and laboratory results. We only later made the suggested change of adding creatinine to the CDS. We were initially resistant to changing this laboratory presentation, as guidelines recommend eGFR—rather than creatinine—as the preferred method for evaluating kidney function [30]. We eventually added creatinine, given the consistent request by end users. Further assessment of usability and uptake of eGFR in practice is warranted. There were also some suggestions that did not result in changes to the CDS. Although 1 suggestion was to list patient medications, we did not choose to do this because of concern related to a conflicting code of *too much information*. We also encountered some suggestions for which we had difficulties with changing the CDS. The biggest limitation in usability was the difficulty with using the order button; problems with this button resulted in some providers not ordering an ACE inhibitor even when they had intended to do so. However, this button was part of the native functionality of the vendor’s EHR alert, and we were advised by our information technology department that the display of the order button was not configurable.

### Limitations

A number of limitations should be considered in the interpretation of the results. First, the study was based in 1 institution and using a single EHR, so results may not be generalizable. Second, usability testing took place in a laboratory setting rather than in the context of the hospital and during a typical workday. As a result, the providers’ experience with usability, and particularly the fit of the CDS tool within their workflow, may not mimic the true clinical setting. Unfortunately, it was not practical to perform usability testing in a true inpatient setting, given the nature of care in the hospital: providers are dealing with multiple patients, dealing with multiple issues, and working in multiple locations at any given time. As a result, hospital-based CDS systems are not triggered at an exact time or place in the workday and, therefore, real-time usability testing is only possible by shadowing providers around for many hours, which was not feasible in the context of this study. To assess usability in clinical practice, we plan to interview providers following implementation; nonetheless, interviews will have to occur after use rather than in real time because of these limitations in working in an inpatient setting. In addition, we plan to quantitatively measure the response rates by all providers for whom these alerts are triggered in a real-world clinical setting. Third, we interviewed a total of 12 providers. This number was based on reaching thematic saturation, and previous studies have suggested 8 to 10 interviews to be sufficient for usability testing [15]. However, our sample was insufficient to determine differences in provider responses by specialty, and our sample may not be representative of providers at NYU Langone Health. Fourth, this study focused on providers who actually place orders in the inpatient system. Attending physicians, although not placing orders, have a significant influence on the care plan and may also benefit from CDS interventions. We hypothesize that attending providers or consultants may have preferences for CDS formats that are similar to supervising residents, although this hypothesis requires further research.

### Conclusions

In one of the first evaluations of comparative usability of interruptive and noninterruptive alerts, we found that there is a trade-off between optimizing visibility and limiting distractions from a current task for interruptive and noninterruptive versions of a CDS. Maximizing the fit of CDS into the workflow is a key element for usability. A potential approach to increase fit into workflow may be to target alert timing and format based on the individual provider role. Whether such an approach leads to an increased uptake in clinical practice needs further evaluation.

## Abbreviations

ACE: angiotensin-converting enzyme
CDS: clinical decision support
EHR: electronic health record
EF: ejection fraction
eGFR: estimated glomerular filtration rate
NP: nurse practitioner
